# Expansion of Mental Health Care in Japanese Obstetric Institutes

**DOI:** 10.7759/cureus.54637

**Published:** 2024-02-21

**Authors:** Shin-ichi Hoshi, Shunji Suzuki, Yoko Sagara, Akihiko Sekizawa, Isamu Ishiwata

**Affiliations:** 1 Obstetrics and Gynecology, Japan Association of Obstetricians and Gynecologists, Tokyo, JPN

**Keywords:** japan, pregnant and postpartum women, mental health problems, obstetric institute, perinatal mental health care

## Abstract

Background: The Japan Association of Obstetricians and Gynecologists (JAOG) has raised awareness of the usefulness of identifying pregnant women with mental health problems and supporting them through multi-professional collaboration. We evaluated the results of questionnaire surveys on mental health care conducted in all obstetric institutes that are members of the JAOG annually.

Methods: Between 2017 and 2023, we requested all obstetric institutes (n = 2,073-2,427) that are members of the JAOG to provide information concerning mental health care for pregnant and postpartum women about the situation in December every year from 2017. Here, we evaluated the results of the questionnaire surveys.

Results: During the study periods, 56.9-74.8% of the 2,073-2,427 institutes responded with valid information. The percentage of obstetric institutes screening for mental health problems during pregnancy and the postpartum period increased from 54.3% and 53.7% to 87.1% and 83.8%, respectively (p < 0.01). However, the proportion of obstetric institutes able to manage pregnant women with mental disorders did not change significantly during the study period.

Conclusion: There has been progress in the active identification of women with mental health problems during pregnancy and the postpartum period. However, the proportion of institutes managing mental disorders has not changed.

## Introduction

For women of reproductive age, mental conditions during pregnancy, childbirth, and child-rearing will change rapidly [1]. Some observations have indicated that symptom levels of depression, anxiety, and/or stress vary over the course of pregnancy [2]. Maternal mental disorders left untreated can lead to several serious social and physical problems, such as suicides including murder-suicide, child abuse, and neglect [3-5]. In addition, perinatal mental disorders have been observed to impair women's functioning and be associated with abnormal child development [3-5]. Therefore, mental health care is necessary for the well-being of pregnant women and their families in all perinatal care settings.

In recent years in Japan, the importance of perinatal mental health care is finally being recognized [6,7]. The Japan Association of Obstetricians and Gynecologists (JAOG) has raised awareness of the usefulness of identifying pregnant women with mental health problems and supporting them through multi-professional collaboration [6,8]. In this study, to examine the effectiveness of the awareness-raising activities of the JAOG, we evaluated the results of questionnaire surveys on mental health care conducted in all obstetric institutes that are members of the JAOG annually.

## Materials and methods

The protocol for this study was approved by the Ethics Committee of the JAOG (No. 20232-2).

Between 2017 and 2023, we requested all obstetric institutes (n = 2,073-2,427) that are members of the JAOG to provide information concerning mental health screening and care for pregnant and postpartum women performed at each obstetric institute around April every year. Eligible institutes were those that manage delivery at the year.

The survey questions requested from each institute were as follows: (i) do you screen for mental health problems during pregnancy, (ii) do you perform the screening for all pregnant women (since 2018), (iii) do you screen for mental health problems in the first month postpartum, (iv) do you perform the screening for all postpartum women (since 2018), (v) are you able to manage pregnant women with mental disorders at your institute, and (vi) do you provide postpartum care services in your institute (since 2018)? The data was collected as yes and no. In this questionnaire survey, the postpartum period was considered up to 12 months after delivery because of the importance of maternal mental health care. Postpartum care services in Japan are those that promote physical recovery and psychological rest for mothers and their children after discharge from delivery obstetric institutes [8].

As shown in Table 1, 56.9-74.8% of the 2,073-2,427 institutes responded with valid information (= the completion of data submitted) during the study periods.

**Table 1:** Number of all obstetric institutes that are members of the Japan Association of Obstetricians and Gynecologists (JAOG), obstetric institutes responding with valid information, and the prevalence of the type of institution (clinic or hospital)

Year	Obstetric institutes	Responses with valid information			
	Total number	Number	Percentage (%)	Percentage of clinics	Percentage of hospitals
2017	2,427	1,382	56.9	-	-
2018	2,374	1,460	61.5	55%	45%
2019	2,330	1,497	64.2	57%	43%
2020	2,282	1,706	74.8	55%	45%
2021	2,188	1,626	74.3	55%	45%
2022	2,146	1,382	64.4	55%	45%
2023	2,073	1,398	67.4	54%	46%

Data are presented as numbers (percentages, %). Differences between the implementation rates of screening and care per year were analyzed using one-way analysis of variance with the statistical software SAS version 8.02 (SAS Institute, Cary, USA). Differences with p < 0.05 were considered significant.

## Results

As shown in Table 1, there was no significant change in the type of obstetric institution, with private clinics (= privately run clinics) accounting for 54-57%.

**Figure 1:**
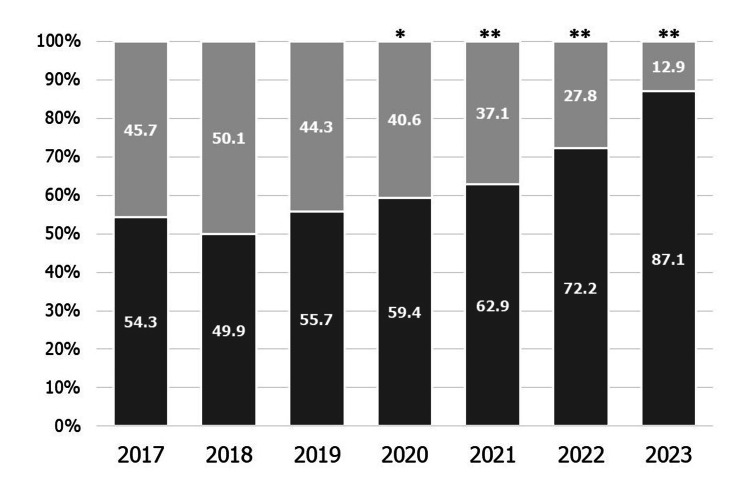
The percentage of obstetrics institutes that screen for mental health problems during pregnancy each year Dark gray: Screening performed. Light gray: Not screened. *p < 0.05 vs. 2017, **p < 0.01 vs. 2017

Figure 1 shows the percentage of obstetrics institutes that screen for mental health problems during pregnancy each year. The percentage of obstetric institutes screening for mental health problems during pregnancy increased from 54.3% to 87.1% (p < 0.01).

**Figure 2:**
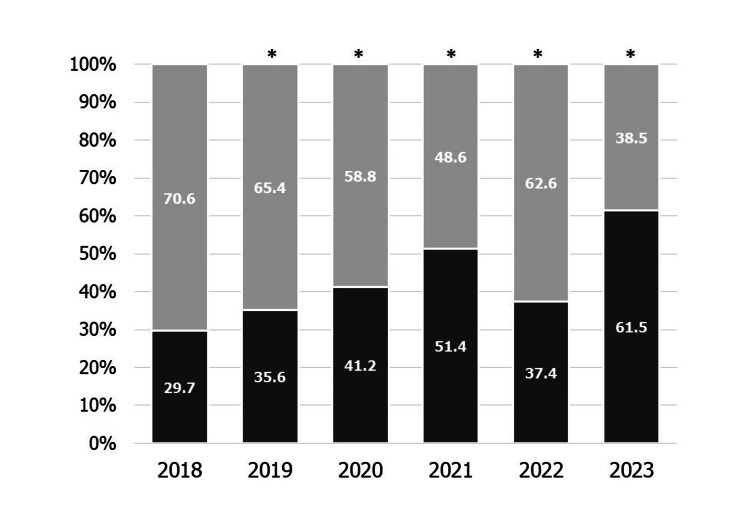
The percentage of obstetric institutes that screen all pregnant women among the institutes screening for mental health problems during pregnancy each year Dark gray: Screen all pregnant women. Light gray: Screen only pregnant women considered as high-risk. *p < 0.01 vs. 2018

Figure 2 shows the percentage of obstetric institutes that screen all pregnant women among the institutes screening for mental health problems during pregnancy each year. The percentage of obstetric institutes screening all women for mental health problems during pregnancy increased from 29.7% to 61.5% (p < 0.01).

**Figure 3:**
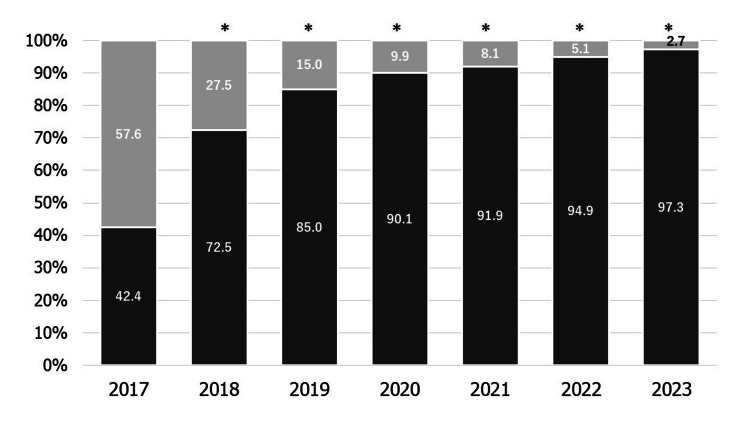
The percentage of obstetrics institutes that screen for mental health problems during the postpartum period each year Dark gray: Screening performed. Light gray: Not screened. *p < 0.01 vs. 2017

Figure 3 shows the percentage of obstetrics institutes that screen for mental health problems during the postpartum period each year. The percentage of obstetric institutes screening for mental health problems during this period increased from 42.4% to 97.3% (p < 0.01). In addition, there were no differences in the mental health screening status of pregnant women in different types of obstetric institutes (p = 0.06).

**Figure 4:**
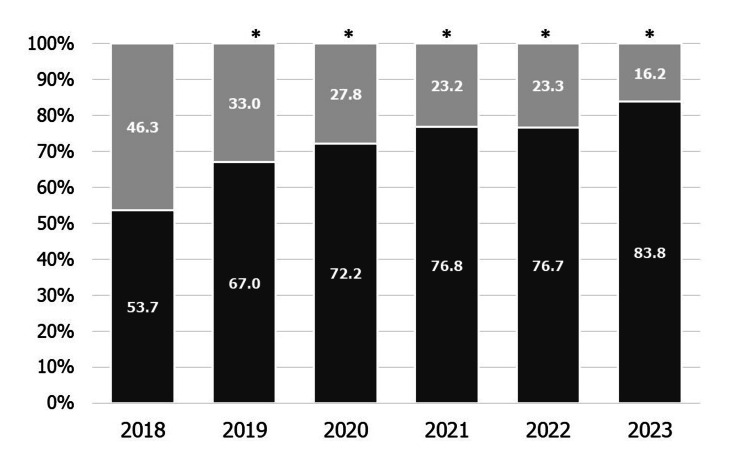
The percentage of obstetric institutes that screen all postpartum women among the institutes screening for mental health problems during the postpartum period each year Dark gray: Screen all postpartum women. Light gray: Screen only postpartum women considered as high-risk. *p < 0.01 vs. 2018

Figure 4 shows the percentage of obstetric institutes that screen all postpartum women among the institutes screening for mental health problems during the postpartum period each year. The percentage of obstetric institutes screening all women for mental health problems during this period increased from 53.7% to 83.8% (p < 0.01).

**Figure 5:**
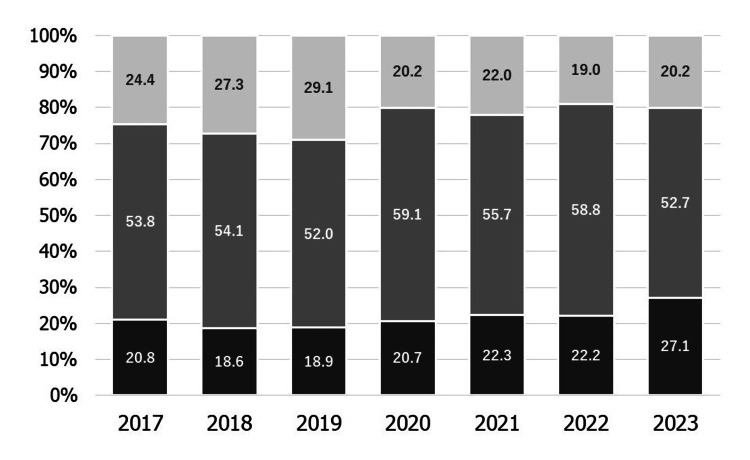
The proportion of obstetric institutes able to manage pregnant women with mental disorders each year Dark gray: Manageable. Moderate gray: Only mild cases can be managed. Light gray: Unmanageable. The results were not statistically significant.

Figure 5 shows the proportion of obstetric institutes able to manage pregnant women with mental disorders each year. The proportion of obstetric institutes able to manage pregnant women with mental disorders did not change significantly during the study period.

**Figure 6:**
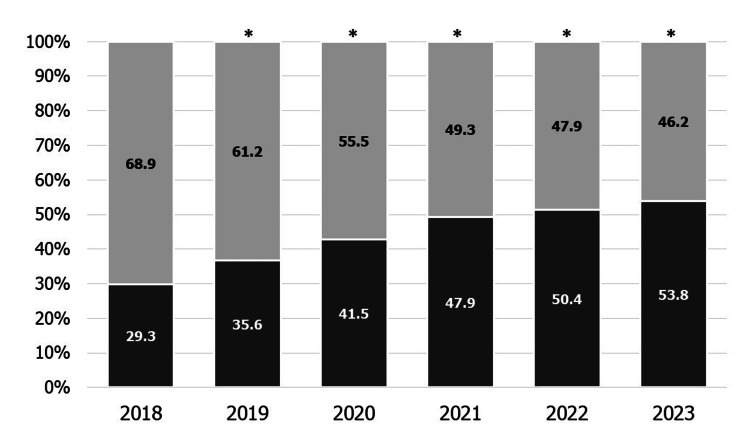
The percentage of obstetric institutes providing postpartum care services each year Dark gray: Provided. Light gray: Not available. *p < 0.01 vs. 2017

Figure 6 shows the percentage of obstetric institutes providing the postpartum care services in each year. The percentage of obstetric institutes providing the postpartum care services increased from 29.3% to 53.8% (p < 0.01).

## Discussion

Based on the current results, in recent years in Japan, there has been progress in the active identification of women with mental health problems during pregnancy and the postpartum period and in support systems for mothers and children with childcare problems, such as postnatal care services [6,7,9,10]. However, unfortunately, it was found that the proportion of obstetric institutes capable of managing pregnant women complicated by mental disorders has not increased.

As shown in Table 1, half of all pregnancies and deliveries are managed in private clinics in Japan, and it has long been a concern that these clinics may not be able to manage pregnancies complicated by mental disorders [11]. Based on the current results, such a concern has not been resolved.

The 2017 edition of Japanese practice guidelines clearly stated higher incidences of depression and anxiety disorders during pregnancy and the postpartum period, and it has since been strongly recommended that the tale of Whooley’s two questions and/or the 2-item Generalized Anxiety Disorder scale (GAD-2) be used to screen a poor mental status in early pregnancy [12-15]. One reason for the change in the guideline description was that suicide with mental disorders was reported to be the most common cause of maternal death during pregnancy and the postpartum period in Japan, as in other developed countries [6]. This has markedly changed our perception as obstetricians, and it also has made us realize that mental health care is as important as, or even more important than, physical care during pregnancy and the postpartum period [7]. Therefore, since 2017, an increasing number of obstetric institutes have screened women for mental health problems during pregnancy and the postnatal period, and an increasing number of institutes appear to have implemented postpartum care services specifically aimed at preventing postnatal depression. Based on these, the JAOG has also raised awareness of the importance of mental health care among the JAOG members, accounting for most of the obstetricians in Japan [6,8], and the current results that revealed these effects are very positive.

On the other hand, the continuing failure of many obstetric institutes to manage pregnant women with moderate or higher-level mental disorders will be one of the key challenges for the perinatal care system in Japan [6,16]. While obstetric management in private clinics contributes to increased comfort for pregnant women based on trust with their chief doctor in Japan, insufficient collaboration with other departments may lead to reduced medical safety. Postpartum care services may contribute to some extent to the prevention of postpartum depression [7]; however, it is also feared that they cannot lead to the treatment of perinatal depression. In Japan, more than half of all deliveries have taken place in private clinics or small hospitals without the presence of pediatricians or psychiatrists [17,18]. Moreover, the current results showed that a small number of obstetric institutes, even if they are hospitals, are unable to manage pregnant women complicated by mental disorders in Japan. Therefore, the current findings may indicate that psychiatrists are under-represented in perinatal mental health care in Japan. In addition, the current findings may indicate that psychiatrists are under-represented in perinatal mental health care in Japan.

In Japan, various guidelines have been published for various occupations according to the level of perinatal mental problems that need to be addressed [7,10]. However, aside from the multidisciplinary collaboration of healthcare workers in obstetric institutions, it will be also necessary to provide opportunities for consultation with psychiatrists.

We understand that there are some serious limitations in this study. First, the surveys had 60-70% response rates, and higher response rates for institutes with a higher level of interest in mental health care. Thus, the current data may not accurately represent the current status of care in Japan. Second, this study examined the changes in the practice arrangements for perinatal mental health care in obstetric institutes; however, it did not examine the consequences of those changes in perinatal outcomes. Third, as the surveys involved obstetricians who are members of the JAOG, it is unclear whether the answers were accurate regarding the medical system of cooperation with psychiatry. Therefore, a long-term study is needed including multiple professions concerning perinatal outcomes to accurately assess the perinatal mental health care system in Japan.

## Conclusions

There has been progress in the active identification of women with mental health problems during pregnancy and the postpartum period in Japan. However, it was found that the proportion of obstetric institutes capable of managing mental disorders has not increased. Therefore, aside from the multidisciplinary collaboration of healthcare workers in obstetric institutions, it will be necessary to provide opportunities for consultation with local administrative staff and psychiatrists.
